# Infantile Metachromatic Leukodystrophy (MLD): A Rare Case

**DOI:** 10.7759/cureus.33155

**Published:** 2022-12-31

**Authors:** Varsha Gajbhiye, Yashwant Lamture, Punam Uke

**Affiliations:** 1 Pediatrics, Jawaharlal Nehru Medical College, Wardha, IND; 2 Surgery, Datta Meghe Institute of Medical Sciences, Wardha, IND

**Keywords:** genetic screening, mental retardation, gait disturbances, autosomal recessive, lipid disorders, white matter

## Abstract

Metachromatic leukodystrophy (MLD) is the typical white matter disease belonging to the lysosomal sphingolipid storage group and is a genetic autosomal recessive disorder. Early presentation is in the form of learning disability and behavioral issues; the subsequent involvement is gait and balance. Prenatal blood testing for genetic screening is available for arylsulfatase A (ARSA) deficiency is indicated if the family history is positive for MLD. Diagnostic tools for MLD are- absence or low-level arylsulfatase activity in genetic screening, sulphatides in urine, and magnetic resonance image (MRI) showing frontal horns and atrial periventricular leukodystrophy. The typical finding is known as the trigonid pattern. A two and half-year-old boy was born out of marriage in near blood relation. No prenatal screening was done. As narrated by the mother, the patient was alright six months back when he gradually developed lower limb weakness. Due to this, he stopped walking, which he could initially do without support. The parent also complained that he used to speak fifteen to twenty words, and now he is not saying a single word. With the above complaint, the patient was taken to the local hospital, where an MRI showed periventricular leukodystrophy, suggesting metachromatic leukodystrophy of periventricular white matter. The practice of prenatal and newborn genetic screening could enhance the efficacy of management, as early interventions are more effective.

## Introduction

In the 1980s, metachromatic leukodystrophies (MLD) were first diagnosed as a progressive disease, mainly affecting the central nervous system's and the peripheral nervous system's myelin sheath and are of genetic origin. No genetic tests were available then, and it remains unrecognizable [1]. The following two decades of development in genetic studies and magnetic resonance imaging (MRI) it leads to the recognization of metachromatic leukodystrophy (MLD) as a genetic anomaly of sulfatide metabolism. Arylsulfatase enzymes present in lysosomes helps in the hydrolysis of sulfatide. The absence of arylsulfatase A (ARSA) enzymes accumulates sulfatide, which damages the myelin in the central nervous and peripheral nervous systems leading to loss of motor and cognitive skills [2].

In the western world, metachromatic leukodystrophy is not a common disorder having an incidence of around from 1/70,000 to 1/90,000. In the Indian subcontinent, it is a rare disorder, as only a few patients are identified to have metachromatic leukodystrophy [3]. Various types of MLD, according to age, are classified as late infantile, juvenile, and adulthood. The infantile form is seen around two years of age or less than that. There is a gradual loss of speech and walking, and the patient does not survive beyond five years of age. Juvenile MLD has been seen between the ages of three and 16. The patient presents with frequent falls, convulsions, loss of muscle control, and difficulty speaking and swallowing, which later ends with a decerebrate posture, and can die ten to twenty years following the onset of the disease. Adult MLD appears in teenagers or adults of any age. Patients may present with difficulty in walking and difficulty in speaking. Diminished vision and loss of hearing, loss of cognition, loss of memory, and abnormal behavior. They generally die within six to fourteen years following the onset of the disease [4].

Accurate and rapid identification is vital for treating this disorder, so genetic screening plays an important role as it is an autosomal recessive disorder with a mutation in the ARSA gene; a family history of arylsulfatase A warrants a genetic screening during pregnancy. Other diagnostic tools for MLD. are the absence of low-sulphatides in urine and MRI [5]. Beside Palliative and supportive treatments, gene therapy and allogeneic hematopoietic stem cell have some hopes of increasing survival rate only if it is done before the onset of neurological symptoms [6].

A handful of cases are identified in India of MLD. There has been a halt in studies on MLD in India, especially on infantile MLD, for a few years. This shows the rarity of its occurrence. Therefore, we report a male child of two and a half years old with the infantile type of MLD, Intending to recognize the features of MLD so that physicians can diagnose and do an early intervention [7].

## Case presentation

**Figure 1:**
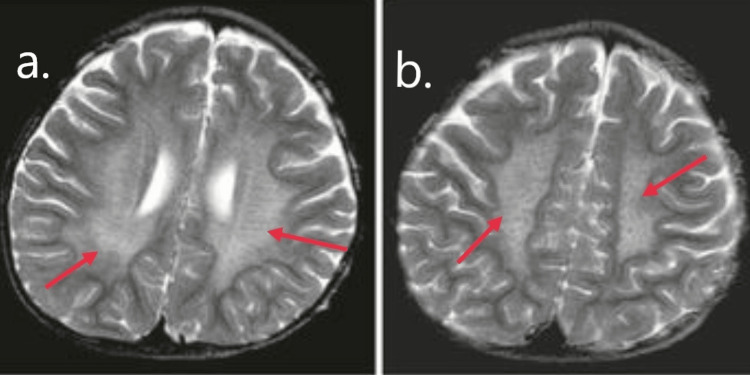
Red arrows in both images a. and b. are of MLD of periventricular white matter These T2-weighted MRI images show symmetrical hyperintensities in bilateral periventricular and cortical white matter is suggestive of Metachromatic leukodystrophy (MLD).

A two-and-a-half-year-old male child was born of second-degree consanguinity marriage. He presented with a history of inability to walk and talk for six months. As narrated by the mother, the patient was alright six months back when he used to walk and run without support but gradually stopped walking and could not walk without support, and could not talk. Developmental history was normal, which included standing with support at 11 months and standing without support at 12 months. By 16 months child could run. Around twenty-four months, he could speak meaningful fifteen to twenty words. He was eating with his hands, using a spoon from a bowl or plate, and could drink from a glass without spilling. Gross motor movement, like standing with support, was affected first, followed by sitting, and later, head control was lost. There is no sign of loss of muscle mass, as seen in muscular dystrophy. The parent also complained of speech loss, where the patient used to speak fifteen to twenty words, and now he does not speak even a single word. It was not associated with fever, seizures, or depressed sensorium. The above-complaint patient was taken to the local hospital, where an MRI was done, suggestive of metachromatic leukodystrophy of periventricular white matter with tigroid pattern (Figure 1). The time duration between hospitalization and diagnosis was about ten days. His birth history was normal. The patient's first cousins had similar complaints and died at five years of age. The primary health center referred the child to our center for further management. The patient was admitted to the pediatric ward. He was vitally stable and was allowed orally. CNS examination showed an increased tone of all four limbs with exaggerated reflexes. Routine complete blood count and serum electrolytes were within normal limits. Genetic testing showed ARSA, homozygous, autosomal recessive, and pathogenic. Urine for sulfatide was recommended but was not done as this facility is not available in the present Institute. The primary healthcare provider referred the patient to a pediatric neurologist, and besides symptomatic treatment, the patient was advised to continue physiotherapy. Tab Baclofen and Syp. levocarnitine was started. The physician explained to the patient that even after doing genetic testing prognosis of the patient would be deteriorating, and no treatment to date is satisfactory. After six months of follow-up, the patient now is in a decerebrate posture, unaware of general surroundings, unable to talk or listen; parents fed with bowl and spoon, and vitals were stable. There is no sign of respiratory failure. He was asked for admission by the medical team, but his parents refused.

## Discussion

Metachromatic leukodystrophy is not a common genetic disease. The prevalence rate of 1 in 40,000-160,000 worldwide, but data on prevalence in India is not found. This disease is because of the high level of sulfatide due to the absence of ARSA enzymes ultimately will damage the myelin of the central and peripheral nervous systems, leading to gradual loss of motor movement, and the patient will have the cognitive disorder. Other related cases of MLD from the literature are depicted as follows (Table 1). A study by Kubaski et al. showed the importance of newborn screening for diagnosing MLD. They have proposed double amniotic fluid screening for sulfatides [8]. This was not similar to the present study. This genetic screening was not done in the present case despite a family history of developmental delay and the death of an only child who was the cousin's brother of the patient and also as it is not routinely recommended in the Indian health setup. In their study, Bindu et al. showed that the association of consanguinity was more commonly seen in MLD, similar to the present study [9]. Convulsions are common manifestations in MLD. Rao et al. showed convulsions as a common symptom observed in infantile MLD., which was not seen in the present study [10].

**Table 1:** Other related cases of MLD from literature. ARSA: arylsulfatase A, MLD: Metachromatic leukodystrophy

Sr.no	Author	conclusion	Present study
1.	Kubaski et al. [8]	Importance of newborn screen for diagnosing MLD	Not done
2	Bindu et al. [9]	association of consanguinity was more commonly seen in MLD	History of consanguinity was present
3	Rao et al. [10]	The confusion associated with MLD	Convulsion not associated
4	Liaw et al. [11]	a survey of MLD showed that the average duration of disease development and loss of motor power to the extent of the bed-ridden vegetative stage was 12 months	In the present case average duration of disease development and loss of motor power to the vegetative stage was within a month
5	Luzi et al. [12]	showed that ARSA was lower in patients with MLD., similar to the present study	Similar to the present case
6	Doherty et al. [13]	depicts the significance of urinary sulfatides and genetic investigation to rule out MLD	Similar to the present case
7	Rastogi et al. [14]	characteristic features of MLD on this imaging modality could alert a confirmatory investigation like ARSA levels in white blood cells	Similar to the present case
8	Shaimardanova et al. [2]	MRI is an informative tool to know the destruction of the periventricular white matter of the brain in MLD	Similar to the present case
9	Fumagalli et al [15]	MLD treatment outcomes are better by gene therapy or allogeneic hematopoietic stem cell but only effective if treatment is given before the development of neurologic symptoms [	Not similar to the present case

The time of progression of the disease varies in each patient. A study done by Liaw et al. in a survey of MLD showed that the average duration of disease development and loss of motor power to the extent of the bed-ridden vegetative stage was 12 months which was not similar to our study. It was half a year in the present case report [11]. In MLD, reduced levels of arylsulfatase A are commonly seen. This is because of the mutation in ARSA genes. Pathologically there will be atrophy of the cerebellum, brainstem, and optic nerves; a decrease in the number of oligodendrocytes. The prognosis for MLD is not good. There is no specific treatment so far. In the infantile form of MLD, most children do not survive after five years, but it can be prolonged with the help of good supportive measures. Luzi et al. showed that ARSA was lower in patients with MLD., similar to the present study [12]. Another study by Doherty et al. depicts the significance of urinary sulfatides and genetic investigation to rule out MLD [13]. In their research, ARSA levels were not elevated. But, urinary sulfatide levels were raised, which was not similar in the present case as in the present study, the patient has a low level of ARSA, and urine for sulfatide was not done as testing facilities are unavailable in the current center.

The importance of MRI in MLD is suggested by the study by Rastogi et al. They concluded that the characteristic features of MLD on this imaging modality could alert a confirmatory investigation like ARSA levels in white blood cells, which were similar to the present study [14]. The survey by Alisa et al. showed that MRI is an informative tool to know the destruction of the periventricular white matter of the brain in MLD. This finding was similar in the present case [2].

Few weapons like bone marrow transplantation and gene therapy are available for the physician to prolong this dreaded genetic disease. However, this is only useful for those who are pre-symptomatic or those with very mild neurological manifestations. This treatment can slow the disease progression and increase the quality of life. In the study, Fumagalli et al. showed MLD treatment outcomes are better by gene therapy or allogeneic hematopoietic stem cell but only effective if treatment is given before the development of neurologic symptoms. CD34+ hematopoietic stem and progenitor cells are collected from the patient's bone marrow. Modified cells are then induced into the gene, which stimulates the ARSA enzyme. In the present case report, a patient came to our center with developed neurological symptoms so gene therapy was not useful [15].

## Conclusions

There have been very few reports of metachromatic leukodystrophies from India. It could also be because of the poor availability of metabolic laboratory and genetic studies facilities. Upcoming pediatric neurology and availabilities of gene screening and metabolic laboratory facilities in every tertiary care will soon be available, bringing hope for these children. Gene therapy and bone marrow transplant can improve survival rates and maintain cognitive and motor function only if done before neurological symptoms develop. So prenatal genetic screening is essential for timely intervention. Now even if there are no specific therapies for MLD in India, correct diagnosis and better supportive management can prevent the complication that can occur due to MLD.
